# Clinical Features and Mortality of Chronic Pulmonary Aspergillosis in Brazil: a Multicenter Cohort Study

**DOI:** 10.1093/ofid/ofaf746

**Published:** 2026-01-13

**Authors:** Vítor Falcão de Oliveira, João Antonio Gonçalves Garreta Prats, Valdes Roberto Bollela, Alessandro Comarú Pasqualotto, Diego Rodrigues Falci, Marcio Nucci, Flavio Queiroz-Telles, Fernanda Guioti Puga, Maria Daniela Bergamasco, Guilherme Santoro-Lopes, Denise Silva Rodrigues, Valério Rodrigues Aquino, Marcello Mihailenko Chaves Magri, David W Denning, Arnaldo L Colombo

**Affiliations:** Division of Infectious Diseases, Hospital das Clínicas da Faculdade de Medicina, University of São Paulo, São Paulo, Brazil; Instituto do Cancer do Estado de Sao Paulo, Faculdade de Medicina da Universidade de Sao Paulo, São Paulo, Brazil; Centres for Antimicrobial Optimisation Network (CAMO-Net), São Paulo, Brazil; Department of Medicine, Division of Infectious Diseases, Escola Paulista de Medicina, Universidade Federal de São Paulo, São Paulo, Brazil; Division of Infectious Diseases, Internal Medicine Department, Hospital das Clínicas da Faculdade de Medicina de Ribeirão Preto, University of São Paulo, São Paulo, Brazil; Universidade Federal de Ciências da Saúde de Porto Alegre, Porto Alegre, Brazil; Santa Casa de Porto Alegre, Porto Alegre, Brazil; University of Minnesota, Minneapolis, USA; Hospital de Clinicas de Porto Alegre, Universidade Federal do Rio Grande do Sul, Porto Alegre, Brazil; Department of Internal Medicine, University Hospital, Federal University of Rio de Janeiro, Rio de Janeiro, Brazil; Oncoclinicas Brazil, Rio de Janeiro, Brazil; Department of Public Health, Federal University of Paraná, Curitiba, Brazil; Division of Infectious Diseases, Internal Medicine Department, Hospital das Clínicas da Faculdade de Medicina de Ribeirão Preto, University of São Paulo, São Paulo, Brazil; Department of Medicine, Division of Infectious Diseases, Escola Paulista de Medicina, Universidade Federal de São Paulo, São Paulo, Brazil; Hospital Israelita Albert Einstein, São Paulo, Brazil; Department of Internal Medicine, Hospital Universitário Clementino Fraga Filho, Universidade Federal do Rio de Janeiro, Rio de Janeiro, Brazil; Instituto Clemente Ferreira, Secretaria de Estado da Saude, São Paulo, Brazil; Hospital de Clinicas de Porto Alegre, Universidade Federal do Rio Grande do Sul, Porto Alegre, Brazil; Division of Infectious Diseases, Hospital das Clínicas da Faculdade de Medicina, University of São Paulo, São Paulo, Brazil; Manchester Fungal Infection Group, Faculty of Biology, Medicine and Health, The University of Manchester, Manchester Academic Health Science Centre, Manchester, UK; Department of Medicine, Division of Infectious Diseases, Escola Paulista de Medicina, Universidade Federal de São Paulo, São Paulo, Brazil; Antimicrobial Resistance Institute of São Paulo-ARIES, São Paulo, Brazil

**Keywords:** chronic pulmonary aspergillosis, mortality, tuberculosis

## Abstract

**Background:**

Data on clinical characteristics and prognosis of chronic pulmonary aspergillosis (CPA) in resource-limited, high tuberculosis (TB) burden settings, especially in Brazil, remain scarce.

**Methods:**

This multicenter retrospective study evaluated all CPA cases diagnosed between 2012 and 2018 across eight centers in Brazil, examining clinical presentation, diagnosis, treatment, mortality, and factors associated with death, including differences related to pulmonary TB. To identify independent predictors of mortality, we conducted multivariate Cox regression. One-year mortality was analyzed using Kaplan–Meier survival curves.

**Results:**

A total of 191 CPA cases were diagnosed, with a median age of 50 years (IQR 40–59) and 62% were male. TB was the most frequent predisposing condition (*n* = 138, 72%). Most patients (80%) received antifungal therapy, primarily itraconazole (*n* = 140, 73%). Surgery was performed in 34% of cases. According to Kaplan–Meier analysis, the cumulative mortality at 12 months was 6%. Compared to survivors, nonsurvivors had significantly lower rates of TB (52% vs 77%, *P* = .019) and higher rates of malignancies (13% vs 3%, *P* = .033). In multivariate analysis, only TB was independently associated with lower mortality (HR 0.413, 95% CI: .179–.954, *P* = .038). The TB group showed more hemoptysis (*P* = .008) and greater radiological involvement, suggesting delayed diagnosis.

**Conclusions:**

In Brazil, the mortality of CPA was lower compared with that reported in previous studies, particularly among patients with TB. In TB-endemic, resource-limited settings, overlapping clinical and radiological features may delay diagnosis and antifungal treatment.

## Teaser

Tuberculosis was independently associated with lower mortality in patients with chronic pulmonary aspergillosis in Brazil.

Chronic pulmonary aspergillosis (CPA) is a progressive and chronic disease of the lungs that typically affects mainly immunocompetent patients with underlying pulmonary comorbidities [1, 2]. Pulmonary tuberculosis (TB) is an important underlying condition among CPA cases [3, 4], but the frequency is variable, based on the prevalence of TB in different countries [5].

The endemicity of TB, especially in low- and middle-income countries, implies an even worse burden of CPA for public healthcare systems. The incidence of TB-associated CPA has been estimated in several countries [5], with approximately 3 million cases worldwide, and it has consequently been recognized as a considerable global health burden [5, 6]. In Brazil, where TB is endemic, the estimated annual incidence of post-TB CPA is 12 032 cases [7].

CPA is linked to significant morbidity and a mortality rate of around 27%, even with antifungal treatment, according to a recent systematic review [8]. However, most CPA studies come from the United Kingdom, South Korea, India, and Japan [9–11]. Clinical features and data on the prognosis of CPA in resource-limited with a high burden of TB settings are scarce, particularly from Brazil, where large case series and prognostic information remain limited [4].

In the present study, we evaluated the clinical presentation, diagnosis, treatment, and mortality of CPA in Brazil, including factors associated with mortality, and provided insights into the differences between patients with and without pulmonary TB as a predisposing condition.

## METHODS

### Design and Setting

This is a multicenter retrospective observational study evaluating all consecutive cases of CPA diagnosed between January 2012 and December 2018 in eight tertiary and quaternary care academic medical centers in southeastern Brazil (comprising over 3000 beds).

### Patient Consent Statement

Informed consent was waived due to the retrospective nature of the study. Ethical approval was obtained from the institutional review boards of all participating centers. All methods were carried out in accordance with relevant national and international ethical guidelines and regulations.

### Definition of CPA

The diagnosis of CPA was based on the updated criteria of the European Society for Clinical Microbiology and Infectious Diseases (ESCMID) and the European Respiratory Society (ERS) guidelines for the management of CPA [12]. All cases required computerized tomography (CT) scan of the chest demonstrating radiological features consistent with aspergillosis, serological and/or microbiological evidence of *Aspergillus* infection, and exclusion of alternative diagnoses.

Patients were further classified into simple aspergilloma (SA), chronic cavitary pulmonary aspergillosis (CCPA)/chronic fibrosing pulmonary aspergillosis (CFPA), *Aspergillus* nodule (AN), and subacute invasive aspergillosis (SAIA) [12]. Additionally, symptoms were required to persist for a minimum of three months, or for at least one month in cases classified as SAIA.

### Data Collection

We developed an online electronic platform with a standardized case report form for all centers, complemented by a dictionary of terms, with the following information: demographic data, past medical history with focus on pulmonary disease and comorbidities, clinical presentation, including respiratory and systemic symptoms, diagnostic methods such as serology, radiology, pathology, and biomarkers, lung function tests, medical and/or surgical therapy and outcomes (mortality within 12 months). Clinical and diagnostic data were collected from medical records prior to the initiation of antifungal therapy. The radiological findings were analyzed based on the reports provided by radiologists from each participating center.

We added the definitions of symptoms, signs, comorbidities, and radiological findings in the Supplementary material (Supplementary Table 1).

### Diagnostic Methods

All patients fulfilled laboratory criteria for CPA by either a single confirmatory test or a combination of positive results such as culture of respiratory samples, detection of specific antibodies against *Aspergillus* spp. (double diffusion or counterimmunoelectrophoresis), galactomannan (GM) in serum and/or bronchoalveolar lavage (BAL) samples, and histology results. Date of diagnosis was defined as the date when patients met all diagnostic criteria.

GM detection was performed by using the Platelia Aspergillus Commercial Enzyme Immunoassay Kit (Bio-Rad Inc.) following manufacturer's instructions. The cutoffs used for positive results were >0.5 and >1.0 for serum and BAL samples, respectively. Identification of *Aspergillus* species was carried out by standard morphological procedures. The antifungal susceptibility test was not performed for most isolates.

Images in chest CT scans were categorized as unilateral or bilateral disease, the presence and number of cavities and surrounding inflammatory signs, consolidation, fibrosis, and presence of aspergillomas.

### Statistical Analysis

All analyses were performed using RStudio (version 1.4) with the *gtsummary* package. Continuous variables were summarized as medians with interquartile ranges (IQR), while categorical variables were presented as frequencies and percentages. We compared patients with and without TB, CPA subtypes and surgical complications, as well as survivors and nonsurvivors. The Mann–Whitney U test was used for continuous variables, and either Fisher's exact test or Pearson's Chi-squared test was applied for categorical variables. To identify independent predictors of mortality, we conducted univariate and multivariate Cox regression. Only two variables of clinical interest (tuberculosis and malignancies) were included due to the limited number of events (*n* = 23), and variables with high collinearity were excluded. Time-to-event data were analyzed using Kaplan–Meier survival curves. The log-rank test was used to compare CPA subtypes and the presence of TB. A *P*-value <.05 was considered statistically significant.

## RESULTS

### Demographic and Clinical Characteristics

A total of 191 cases of chronic pulmonary aspergillosis (CPA) were diagnosed. Table 1 summarizes the main characteristics of these patients. The median age was 50 years, and the majority of the patients were male (62%). The most frequent pulmonary predisposing conditions for CPA were TB (72%), particularly post-TB sequelae (*n* = 128/138, 93%). Only 10 cases (7%) were active TB. Bronchiectasis was present in 70% of patients, and in 80% of those cases (107/134), it was associated with TB. Smoking was reported in 54% and alcohol consumption in 30%.

**Table 1.** Demographic, Clinical, and Radiological Characteristics of 191 Patients With Chronic Pulmonary Aspergillosis

Characteristics	Number (%)
Age (years), median (IQR)	50 (40–59)
Male gender	118 (62)
Pulmonary conditions	
Tuberculosis	138 (72)
Bronchiectasis	134 (70)
COPD	41 (21)
Thoracic surgery	20 (10)
Nontuberculous mycobacteriosis	13 (7)
Asthma	11 (6)
Lung cancer	3 (2)
Comorbidities	
Smoking	104 (54)
Alcohol consumption	57 (30)
Diabetes mellitus	28 (15)
HIV infection	18 (9)
Malignancies	9 (5)
Solid organ transplant	7 (4)
Signs and symptoms	
Cough	164 (86)
Dyspnea	139 (73)
Productive cough	125 (65)
Hemoptysis	122 (64)
Weight loss	84 (44)
Fever	42 (22)
Chest pain	40 (21)
Radiological findings	
Cavities	175 (92)
Two or more cavities	93 (53)
Inflammatory signs surrounding cavities	113 (65)
More than 1 affected lung lobe	162 (85)
Aspergilloma	155 (81)
Fibrosis/architectural distortion	148 (77)
Bilateral involvement	146 (76)
Consolidation	104 (54)

Abbreviations: IQR, interquartile range; COPD, chronic obstructive pulmonary disease.

A minority were immunosuppressed patients. There were seven cases of solid organ transplantation: three lung transplants, and two each of liver and kidney transplants. 15% of patients had diabetes mellitus.

Cough was reported in 164 patients (86%), of whom 125 (65%) had a productive cough. Other common clinical manifestations included dyspnea (73%) and hemoptysis (64%). Fever (22%) and chest pain (21%) were less frequently observed.

### Diagnostic Characteristics

The most frequent imaging findings in chest CT scans were cavities (92%), aspergillomas (81%), and fibrosis and architectural distortion (77%). Bilateral lung involvement was present in 76% of cases, affecting more than one lung lobe (85%).

Most patients were classified as having CCPA/CFPA (63%), followed by simple aspergilloma (25%) and SAIA (10%) (Table 2). *Aspergillus* nodules were identified in only three cases. When available, lung function test results showed mostly obstructive lung disease (66%), and most patients had severe disease (56%).

**Table 2.** Classification, Lung Function Tests, and Diagnosis in Patients With Chronic Pulmonary Aspergillosis

Characteristics	Number (%)
CPA subtype	
CCPA or CFPA	121 (63)
Simple aspergilloma	47 (25)
SAIA	20 (10)
*Aspergillus* nodule	3 (2)
Lung function test result	
Obstructive	31/47 (66)
Restrictive	9/47 (19)
Nonspecific^a^	7/47 (15)
Lung function test classification	
Severe (FEV_1_ ≤ 40%)^b^	25/45 (56)
Moderate (FEV_1_ 41–59%)	16/45 (35)
Mild (FEV_1_ ≥ 60%)	4/45 (9)
Diagnostic test—no. positive	
Culture	71/172 (41)
*Aspergillus* antibodies by ID/CIE	103/143 (72)
Serum GM	16/52 (31)
Pathology	65/78 (83)
BAL GM	26/35 (74)
Etiology (*Aspergillus s*pecies complex, *N* and %)	
*Aspergillus fumigatus*	28/68^c^ (41)
*Aspergillus flavus*	5/68 (7)
*Aspergillus niger*	4/68 (6)
*Aspergillus* spp.	31/68 (46)

Abbreviations: CPA, chronic pulmonary aspergillosis; CCPA, chronic cavitary pulmonary aspergillosis; CFPA, chronic fibrosing pulmonary aspergillosis; SAIA, subacute invasive pulmonary aspergillosis; GM, galactomannan.

^a^The test showed abnormalities but did not fit criteria for obstruction or restriction.

^b^FEV_1_, forced expiratory volume in 1 s (% predicted)—for obstructive patterns.

^c^Number of cases for which the information was available.

The diagnostic tests with the highest positivity rates were: pathology (83%), BAL GM (74%), and serology (72%). Culture and serum GM showed lower positivity rates (41% and 31%, respectively). Nearly, 50% of cases did not yield species isolation. Among the cultures with species identified, *Aspergillus fumigatus* was the most frequent (76%).

### Treatment

Most patients received antifungal therapy (80%) (Table 3). Itraconazole was the most commonly used antifungal (73%), while voriconazole was used less frequently (19%). Among patients with CPA and active TB, two received amphotericin B, four were treated with itraconazole, and four received no antifungal therapy as priority was given to TB treatment. Surgical treatment was performed in 34% of cases, with lobectomy being the most common procedure. The main indication for surgery was hemoptysis (58%), and bronchial artery embolization was performed in 13% of patients. Surgical complications were frequent, affecting 62% of patients, with empyema and bleeding being the most prevalent. CCPA/CFPA (77%, 24/31) and SAIA (80%, 8/10) were the most common subtypes associated with surgical complications, while SA (35%, 8/23) and AN (0%) were the least frequent, with statistically significant differences (*P* = .002).

**Table 3.** Management and Mortality of 191 Patients With Chronic Pulmonary Aspergillosis

Characteristics	Number (%)
Antifungal therapy	
Any antifungal	152 (80)
Itraconazole	140 (73)
Voriconazole	37 (19)
Amphotericin B formulation	12 (6)
Echinocandin	2 (1)
Bronchial artery embolization	24/183 (13)
Lung surgery	65 (34)
Lobectomy	36 (55)
Pneumonectomy	14 (22)
Segmentectomy	13 (20)
Other	2 (3)
Indication for surgery	
Hemoptysis	38 (58)
Localized disease	21 (32)
Clinical failure	4 (6)
Diagnosis uncertain	2 (3)
Postoperative complications	
Any	40/65 (62)
Empyema	15/40 (38)
Bronchopleural fistula	8/40 (20)
Bleeding	11/40 (28)
Respiratory failure	5/40 (13)
Pneumonia	6/40 (15)
Death	4/40 (10)
Overall 1-year mortality	23 (13)

### Mortality

**Figure 1.**
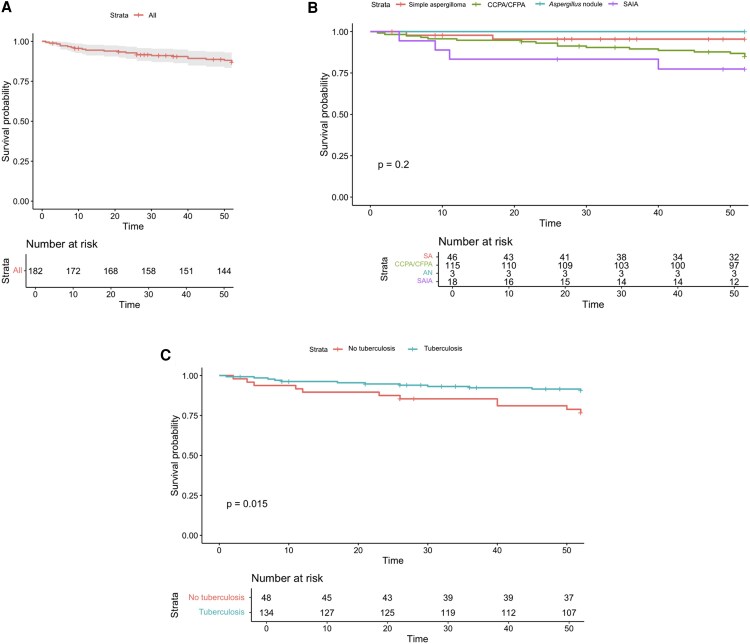
Kaplan–Meier survival estimates. (A) All patients. (B) CPA subtypes. (C) Tuberculosis. Abbreviations: CCPA, chronic cavitary pulmonary aspergillosis; CFPA, chronic fibrosing pulmonary aspergillosis; SAIA, subacute invasive pulmonary aspergillosis; SA, simple aspergilloma; AN, *Aspergillus* nodule.

The Kaplan–Meier estimated cumulative mortality was 6% at 12 months (Figure 1). TB was associated with lower mortality (log-rank *P* = .015). One death occurred among patients with CPA and active TB coinfection. No statistically significant differences were observed among the different CPA subtypes (log-rank *P* = .200).

The nonsurvivor group had statistically significantly lower TB (*P* = .019) and bronchiectasis (*P* = .008) diagnosis, and higher malignancies (*P* = .033). The comparison between survivors and nonsurvivors, as well as the multivariate analysis of factors associated with mortality, is summarized in Table 4. In the multivariate model, only TB (HR 0.413; 95% CI: .179–.954; *P* = .038) was independently associated with a lower risk of mortality. Bronchiectasis was excluded from analysis due to its high collinearity with TB, and amphotericin B was excluded due to its high collinearity with mortality.

**Table 4.** Comparison Between Survivor and Nonsurvivor Groups of Patients With Chronic Pulmonary Aspergillosis Checking for Variables Associated With Mortality by Multivariate Analysis

Characteristics *N* = 191	Survivor *N* = 159	Nonsurvivor *N* = 23	*P-*Value	Multivariate Analysis		
				HR	95% CI	*P*-value
Age median, years (IQR)	50 (40–59)	58 (41–69)	.101	…	…	
Male	96 (60%)	15 (65%)	.634	…	…	
Underlying conditions						
Tuberculosis	122 (77%)	12 (52%)	.019	0.413	.179–.954	.038
Nontuberculous mycobacteriosis	11 (7%)	1 (4%)	.589	…	…	
DPOC	33 (21%)	6 (26%)	.606	…	…	
Asthma	8 (5%)	2 (9%)	.491	…	…	
Bronchiectasis	120 (75%)	11 (48%)	.008	…	…	
Lung cancer	2 (1%)	1 (4%)	.276	…	…	
Thoracic surgery	15 (9%)	4 (17%)	.255	…	…	
Diabetes mellitus	24 (15%)	4 (17%)	.751	…	…	
Malignancies	5 (3%)	3 (13%)	.033	2.849	.825–9.839	.097
Solid organ transplant	5 (3%)	1 (4%)	.833	…	…	
HIV infection	15 (9%)	3 (13%)	.686	…	…	
Alcohol use	45 (28%)	9 (39%)	.252	…	…	
Smoking	84 (53%)	15 (65%)	.306	…	…	
Radiological findings						
More than 1 affected lung lobe	136 (86%)	19 (83%)	.612	…	…	
Bilateral disease	122 (77%)	17 (74%)	.664	…	…	
Cavitation	146 (92%)	21 (91%)	.968	…	…	
Two or more cavities	79 (50%)	12 (52%)	.852	…	…	
Aspergilloma	131 (82%)	18 (78%)	.765	…	…	
Inflammatory signs surrounding cavities	91 (57%)	16 (70%)	.287	…	…	
Consolidation	83 (52%)	13 (57%)	.769	…	…	
Fibrosis with architectural distortion	119 (75%)	22 (96%)	.066	…	…	
Mycological diagnosis						
Positive serum GM	12 (29%)	1 (14%)	.459	…	…	
Positive BAL GM	19 (73%)	3 (60%)	.450	…	…	
Positive serology	91 (73%)	10 (67%)	.630	…	…	
Positive culture	59 (41%)	9 (47%)	.576	…	…	
Etiology (species complex)						
*Aspergillus flavus*^a^	2 (8%)	2 (22%)	-	…	…	
*Aspergillus fumigatus*	20 (80%)	7 (78%)	.231	…	…	
*Aspergillus niger*	3 (12%)	0 (0%)	-^b^	…	…	
Clinical forms of CPA			.130			
*Simple aspergilloma*	44 (28%)	2 (9%)	.055	…	…	
*CCPA/CFPA*	98 (62%)	17 (74%)	.364	…	…	
*Aspergillus nodule*	3 (2%)	0 (0%)	-^b^	…	…	
*SAIA*^a^	14 (9%)	4 (17%)	-	…	…	
Treatment strategies						
Itraconazole	115 (72%)	17 (74%)	.898	…	…	
Voriconazole	30 (19%)	5 (22%)	.781	…	…	
Echinocandins	1 (1%)	1 (4%)	.135	…	…	
Amphotericin B	7 (4%)	5 (22%)	.001	**…**	**…**	
Surgery	54 (34%)	9 (39%)	0.521	…	…	
Surgical complications	31 (57%)	8 (89%)	.117	…	…	

Abbreviations: IQR, interquartile range; HR, hazard ratio; COPD, chronic obstructive pulmonary disease; CPA, chronic pulmonary aspergillosis; CCPA, chronic cavitary pulmonary aspergillosis; CFPA, chronic fibrosing pulmonary aspergillosis; SAIA, subacute invasive pulmonary aspergillosis.

^a^Reference.

^b^The number of patients was zero in the nonsurvivor group.

### Comparison of CPA Patients With and Without TB

When comparing patients with and without TB, a similar median age was observed (*P* = .500) (Table 5). There was a higher proportion of male patients in the TB group (67% vs 49%, *P* = .025), along with a significantly greater frequency of bronchiectasis (80% vs 45%, *P* < .001) and alcohol use (35% vs 17%, *P* = .016). Conversely, lung cancer and solid organ transplantation were less frequent in the TB group. The clinical presentation was generally similar between groups; however, hemoptysis was significantly more frequent in the TB group (70% vs 49%, *P* = .008). Regarding radiological findings, involvement of more than one lung lobe (89% vs 74%, *P* = .007), presence of two or more cavities (54% vs 36%, *P* = .028), fibrosis (82% vs 66%, *P* = .019), aspergilloma (86% vs 70%, *P* = .013), and bilateral disease (80% vs 66%, *P* = .036) were significantly more frequent in patients in the TB group. The TB group had higher frequencies of simple aspergilloma, CCPA, and CFPA, while SAIA was significantly more prevalent in the non-TB group (21% vs 7%, *P* = .009). Postoperative complications were similar between the groups (73% vs 58%; *P* = .300). Lastly, significantly lower mortality in the non-TB group compared to the TB group (9% vs 23%, *P* = .012) was observed.

**Table 5.** Comparison Between Tuberculosis and Nontuberculosis Groups in Patients With Chronic Pulmonary Aspergillosis

Characteristics *N* = 191	Non-TB *N* = 53	TB *N* = 138	*P*-Value
Age median, years (IQR)	56 (39–66)	50 (40–59)	.500
Male gender	26 (49%)	92 (67%)	.025
Comorbidities			
Nontuberculous mycobacteriosis	6 (11%)	7 (5%)	.200
Smoking	26 (49%)	78 (57%)	.400
Alcohol use	9 (17%)	48 (35%)	.016
Thoracic surgery	11 (21%)	9 (7%)	.004
COPD	9 (17%)	32 (23%)	.300
Asthma	4 (8%)	7 (5%)	.500
HIV	2 (4%)	16 (12%)	.200
Diabetes mellitus	11 (21%)	17 (12%)	.140
Bronchiectasis	24 (45%)	110 (80%)	<.001
Malignancies	5 (9%)	4 (3%)	.120
Lung cancer	3 (6%)	0 (0%)	.020
Solid organ transplant	7 (13%)	0 (0%)	<.001
Signs and symptoms			
Cough	47 (89%)	117 (85%)	.500
Productive cough	35 (66%)	90 (65%)	>.900
Dyspnea	36 (68%)	103 (75%)	.400
Hemoptysis	26 (49%)	96 (70%)	.008
Weight loss	22 (42%)	62 (45%)	.700
Fever	14 (26%)	28 (20%)	.400
Chest pain	9 (17%)	31 (22%)	.400
Radiological findings			
More than 1 affected lung lobe	39 (74%)	123 (89%)	.007
Fibrosis with architectural distortion	35 (66%)	113 (82%)	.019
Cavities	45 (85%)	130 (94%)	.075
Two or more cavities	19 (36%)	74 (54%)	.028
Aspergilloma	37 (70%)	118 (86%)	.013
Bilateral disease	35 (66%)	111 (80%)	.036
Inflammatory signs surrounding cavities	29 (55%)	84 (61%)	.400
Consolidation	32 (60%)	72 (52%)	.300
CPA subtypes			.009
CCPA or CFPA	31 (58%)	90 (65%)	
Simple aspergilloma	9 (17%)	38 (28%)	
SAIA	11 (21%)	9 (7%)	
*Aspergillus* nodule	2 (4%)	1 (1%)	
Postoperative complications	11/15 (73%)	29/50 (58%)	.300
Overall 1-year mortality	11/48 (23%)	12/134 (9%)	.012

Abbreviations: TB, tuberculosis; IQR, interquartile range; COPD, chronic obstructive pulmonary disease; CCPA, chronic cavitary pulmonary aspergillosis; CFPA, chronic fibrosing pulmonary aspergillosis; SAIA, subacute invasive pulmonary aspergillosis.

## DISCUSSION

We present a large multicenter retrospective cohort study of CPA from Brazil, highlighting the importance of TB as an underlying condition, accounting for over 70% of cases. The cumulative mortality at 12 months was 6%. Patients with TB more frequently exhibited bronchiectasis, hemoptysis, and extensive radiological findings, yet showed lower mortality compared to those with other comorbidities.

The exact global burden of CPA among patients treated for TB remains unclear; however, a recent systematic review reported a prevalence of 9% among all TB patients and 48% among those with persistent symptoms evaluated after completing treatment [13]. Similar to the cohorts from Pakistan, the Brazilian cohort exhibited a higher proportion of TB cases compared to other regions of the world, as well as a considerable proportion of patients with diabetes mellitus [4, 8, 14–17]. Furthermore, we observed a higher proportion of bronchiectasis in our study, likely related to TB, which is considered is commonly associated with CPA [18]. These findings underscore the need for greater clinical awareness to distinguish CPA from other respiratory diseases with overlapping, nonspecific symptoms, especially in TB-endemic regions where it is often underdiagnosed [19, 20]. As most patients had bilateral involvement and severe disease, this may indicate a delay in diagnosis, although the interval between symptom onset and diagnosis was not assessed.

In our study, pulmonary symptoms such as cough, dyspnea, and expectoration were more common—particularly hemoptysis in the TB group—while systemic symptoms like fever were less frequent, consistent with findings in the literature [21, 22]. In settings with a high burden of TB, patients who return with new or persistent respiratory symptoms should be evaluated not only for TB recurrence but also for the possibility of CPA, making it essential to perform specific diagnostic tests to confirm the diagnosis. The single most important investigation test is a serum *Aspergillus* IgG assay, positive in 70–90% of patients depending on the study [23, 24]. In addition, there is a wider differential fungal diagnosis including chronic cavitary pulmonary histoplasmosis, coccidioidomycosis, and paracoccidioidomycosis [25], all with elevated IgG antibody to the causative fungus.

Our data demonstrated that histology, GM in BAL fluid, and *Aspergillus* antibodies by CIE/ID were the most accurate diagnostic tests, with positivity rates exceeding 70%, closely aligning with previously reported findings [26, 27]. However, GM in BAL and histology are more invasive methods and are typically reserved for cases in which serology, a noninvasive test, fail to establish a diagnosis [28–30]. All serological testing in our study was precipitin-based, with no use of anti-*Aspergillus* IgG ELISA, despite its superior accuracy [24]. Additional cases of CPA might have been diagnosed with a more sensitive assay, an issue for many working in resource-limited settings [31].

Although serum GM shows high specificity for CPA, its sensitivity is relatively low, and it is generally not recommended for routine diagnostic use [32, 33]. However, in cases of moderate immunosuppression, such as in patients with HIV infection or rheumatologic diseases with possible SAIA, serum GM does exhibit higher sensitivity due to a pathophysiological process resembling that of acute invasive aspergillosis [12, 27, 34]. Additionally, cultures from respiratory samples showed low positivity rates (41%), likely due to the collection of a single specimen for diagnosis or the use of sample volume being divided among multiple tests. Recent studies have shown that the recovery rate of *Aspergillus* spp. is significantly higher when using high-volume culture techniques compared to conventional methods [35].

A recent systematic review reported an overall 1-year mortality of 15%, higher than our findings, and identified a history of pulmonary TB as being associated with the lowest mortality risk, whereas the highest mortality rates were observed in patients with underlying malignancies and SAIA [8, 36]. Although the TB group exhibited radiological features suggestive of more extensive pulmonary parenchymal involvement in our study, the lower mortality rates may be attributed to a lower proportion of SAIA and fewer patients with immunosuppressive conditions. Additionally, the radiological findings may reflect residual sequelae rather than active disease, with no indication of ongoing progression. We did not observe differences between all CPA subtypes. However, the small sample size limited definite conclusions between CPA subtypes.

Furthermore, most cases in our cohort appeared to represent advanced disease, as evidenced by the presence of fibrosis in 77% of patients and severe impairment on lung function tests (56%). Since fibrosis represents the final stage in the natural history of CPA [12], this observation implies that the diagnosis was made late in the course of CPA in many cases. Additionally, patients with interstitial lung disease, which encompasses many conditions with pulmonary fibrosis as a central component, also had high mortality, accounting for 42% [8], although only a few patients in our cohort had a previous diagnosis of interstitial lung disease.

Our study has several limitations. First, we reported only a 1-year follow-up period, which may underestimate the mortality rate as a significant number of deaths occurs up to 5 years in CPA. Another limitation is the heterogeneity in access to diagnostic tests and treatment options between centers. For example, the restricted availability of diagnostic tools may have contributed to significant missing data related to the diagnosis of CPA and lack of identification of some cases. Moreover, data on azole resistance and *Aspergillus* species identification were not available—both of which could be factors that may influence mortality. Of note, the exact duration of CPA symptoms was uncertain in most cases due to the retrospective design. Furthermore, we cannot exclude the possibility that fibrosis was already present before CPA diagnosis (eg, during active or post-TB stages), which may have influenced our finding of a high prevalence of fibrosis. Lastly, information on the completion of antituberculous therapy and the timing of CPA diagnosis or adverse events were not assessed.

## CONCLUSION

In Brazil, the mortality of CPA was lower compared with that reported in previous studies, particularly among patients with TB. In resource-limited settings with a high burden of TB, overlapping clinical and radiological features may delay diagnosis of CPA and the initiation of antifungal therapy, resulting in a high prevalence of fibrosis.

## Supplementary Material

ofaf746_Supplementary_Data

## Supplementary Data

Supplementary materials are available at *Open Forum Infectious Diseases* online. Consisting of data provided by the authors to benefit the reader, the posted materials are not copyedited and are the sole responsibility of the authors, so questions or comments should be addressed to the corresponding author.
