# Supplementary material for: Clinical Features and Mortality of Chronic Pulmonary Aspergillosis in Brazil: a Multicenter Cohort Study
Source: Open Forum Infect Dis. 2026 Jan 13;13(1):ofaf746. doi: 10.1093/ofid/ofaf746 (PMC12798536; doi:10.1093/ofid/ofaf746)
Supplement: ofaf746_Supplementary_Data [file ofaf746_supplementary_data.docx]

**Supplementary Material**

**Oliveira VF *et al*.** Clinical features and mortality of chronic pulmonary aspergillosis in Brazil: a multicenter cohort study

| Table S1 | Pages 2-3 |
| --- | --- |

**Table S1.** Data dictionary: definitions of symptoms, signs, comorbidities, and radiological findings

| **Symptoms and signs** | **Definition** |
| --- | --- |
| Cough | Presence of persistent cough |
| Dyspnea | Subjective sensation of shortness of breath of any severity |
| Productive cough | Cough associated with expectoration of sputum |
| Hemoptysis | Expectoration of blood or blood-streaked sputum |
| Weight loss | Unintentional decrease in body weight |
| Fever | Body temperature ≥ 37.8 °C documented by measurement or reported subjectively by the patient |
| Chest pain | Thoracic pain, regardless of intensity or duration |
| **Comorbidities** |  |
| Tuberculosis | Pulmonary tuberculosis was defined by the presence of at least one positive microbiological criterion, including acid-fast bacilli smear, rapid molecular test, and/or mycobacterial culture |
| Active tuberculosis | Patients receiving antituberculous treatment at the time of CPA diagnosis |
| Post-tuberculosis sequelae | Patients with a documented history of tuberculosis with completed treatment, or radiological sequelae compatible with prior disease |
| Thoracic surgery | History of any surgical procedure involving the lungs, pleura, or mediastinum |
| Nontuberculous mycobacteriosis | Documented diagnosis of pulmonary caused by nontuberculous mycobacteria, confirmed by microbiological criteria |
| Bronchiectasis | Documented diagnosis of irreversible bronchial dilatation confirmed by chest computed tomography scan |
| Asthma | History of asthma documented in medical records |
| Chronic obstructive pulmonary disease | History of chronic obstructive pulmonary disease documented in medical records |
| Lung cancer | Any primary malignant neoplasm of the lung |
| Malignancies | Solid or hematological cancer |
| Diabetes mellitus | History of diabetes mellitus documented in medical records |
| HIV infection | History of HIV infection documented in medical records |
| Solid organ transplant | Documented history of transplantation of a solid organ (e.g., kidney, liver, heart, or lung) |
| Smoking | Any history of tobacco smoking, regardless of amount, frequency, or duration, past or present |
| Alcohol consumption | Any history of alcohol consumption, regardless of amount, frequency, or duration, past or present |
| **Radiological findings #** |  |
| Fibrosis | Architectural distortion of lung parenchyma associated with volume loss, honeycombing, or traction bronchiectasis |
| Consolidation | Homogeneous increase in pulmonary parenchymal attenuation that obscures the margins of vessels and airway walls. |
| Cavitation | Gas-filled space within consolidation, a mass, or a nodule, resulting from expulsion of necrotic material |
| Aspergilloma | Intracavitary mass (fungal ball) within a pre-existing lung cavity |
| **# Reference:** Hansell DM, Bankier AA, MacMahon H, McLoud TC, Müller NL, Remy J. Fleischner Society: glossary of terms for thoracic imaging. *Radiology*. 2008;246(3):697–722. | |
